# Whole Body Vibration Exposure Transmitted to Drivers of Heavy Equipment Vehicles: A Comparative Case According to the Short- and Long-Term Exposure Assessment Methodologies Defined in ISO 2631-1 and ISO 2631-5

**DOI:** 10.3390/ijerph19095206

**Published:** 2022-04-25

**Authors:** María L. de la Hoz-Torres, Antonio J. Aguilar, Diego P. Ruiz, Mª Dolores Martínez-Aires

**Affiliations:** 1 Department of Building Construction, University of Granada, Av. Severo Ochoa s/n, 18071 Granada, Spain; mlhoz@ugr.es (M.L.d.l.H.-T.); aires@ugr.es (M.D.M.-A.); 2 Department of Applied Physics, University of Granada, Av. Severo Ochoa s/n, 18071 Granada, Spain; druiz@ugr.es

**Keywords:** whole-body vibration, ISO2631-1:1997, ISO2631-5:2018, Heavy equipment vehicles

## Abstract

The construction and transport sectors are the industries with the highest proportions of workers exposed to vibrations in the European Union. Heavy equipment vehicle (HEV) drivers often perform operations on different uneven surfaces and are exposed to whole body vibration (WBV) on a daily basis. Recently, a new version of ISO 2631-5 was published. However, since this new method required as input the individual exposure profile and the acceleration signals recorded on more surfaces, limited studies have been carried out to evaluate HEV operations according to this standard. The objectives of this study were to assess the WBV exposure using the methods defined in ISO 2631-1:1997 and ISO 2631-5:2018 and to compare the obtained health risk assessments between drivers with different anthropometric characteristics. For this purpose, two drivers were selected and a field measurement campaign was conducted. Regarding short-term assessment, results showed that VDV was the most restrictive method with exposure levels above the exposure action limit value, while $S_{d}^{A}$ indicated that the same exposures were safe for the worker. With respect to long-term assessment, Risk Factor R^A^ showed that the driver with the highest body mass index was the only one who exceeded the low probability limit of adverse health effects.

## 1. Introduction

The second European Survey of Enterprises on New and Emerging Risk (ESENER-II) revealed that 70.9% of workers claim to be regularly exposed to physical agents at their workplace [1]. Among these agents, mechanical vibration is one of the physical risks to which workers are commonly exposed. However, statistically significant differences were found among sectors in relation to this exposure, with the highest proportions of workers exposed to vibration at least one quarter of the time being in the Construction and Transport sector (45%) and in the Agriculture and Industry sector (42%) [2].

In these industries, driving heavy equipment vehicles (HEV) exposed workers to whole-body vibrations (WBV) on a daily basis [3,4,5,6,7]. Vibrations are mainly transmitted to the driver’s body through the vehicle seat and the cabin floor [8,9]. In fact, since HEV drivers often perform activities on different types of surface (e.g., off-road, unpaved road, tarmac road, etc.), they are frequently exposed to higher levels of WBV and mechanical shocks [10,11,12]. The WBV exposure may last for long periods of time (6–7 h) during operations such as transport of materials, earth-moving, demolition processes, etc., and, therefore, it may involve a high risk to the worker’s health [13].

Several epidemiological studies have evidenced a relationship between long-term exposure to WBV and the occurrence of musculoskeletal disorders (MSDs), such as low back pain [14,15,16], sciatica [17], neck pain [18,19], degenerative changes in the lumbar spine [20], and other disorders such as cognitive/motor impairment. In fact, Costa et al. [21] found evidence of cognitive/motor impairment as a result of WBV exposure. MSDs are a cause of concern due to their impact on individual workers’ health, the social costs to European countries and the economic impacts on the enterprises [22]. Indeed, the first finding report published by the European Agency for Safety and Health at Work (EU-OSHA) about the ESENER 2019 reveals that MSDs are one of the biggest concerns for European workplaces [23,24].

In this context, the European Union defined minimum levels of worker protection to WBV exposure in order to control this risk in the workplace. Directive 2002/44/EC [25] states that the method to be used for the assessment of WBV exposure is the daily exposure A(8) or the highest vibration dose value (VDV), which are defined in the ISO 2631-1:1997 standard [26]. Directive 2002/44/CE also lays down the daily exposure action value (EAV) and the daily exposure limit value (ELV) for WBV exposure. However, the risk assessment in both methods and the limit values are limited to short-term exposure (over a day) and do not consider the cumulative effects of long-term WBV exposure. Since previous research has highlighted that cumulative exposure to WBV is one of the leading risks for the development of low back disorders [27,28], safety managers should be mindful of this constraint. Furthermore, the recent study conducted by Bovenzi et al. [29] pointed out that there is some evidence that the EU exposure limit values are excessive; so much so that WBV exposure beneath these limit values is associated with an elevated risk of low back pain. In addition, drivers with high body mass index (BMI) belong to the high-risk group, since previous research found that overweight and obesity increasing the risk of lumbar disc degeneration [30,31].

Nevertheless, there are other methods defined in ISO standards that can be applied in a complementary manner for the assessment of WBV exposure. In this case, since driving HEVs involves exposure to vibrations containing multiple shocks, the method defined in ISO 2631-5:2004 [32] may be considered as a complementary method to specifically assess this type of exposure. In contrast to ISO 2631-1, the method described in this standard includes the Factor R, which is used to assess the adverse health effects of long-term whole-body multiple-shock exposure and is calculated sequentially, taking into account the increasing age (and reducing strength) of the worker as the exposure time increases. This standard has recently been revised and the ISO 2631-5:2018 [33] has been released. This new version of the standard defines assessment methods that also include the posture and the anthropometric variables of the driver.

Previous research has studied WBV exposure according to the methods defined in ISO 2631-1:1997 and ISO 2631-5:2004 [34,35,36,37,38]. Indeed, Eger et al. [39] pointed out a discrepancy regarding the presence of health risks and that the health guidance boundaries stated in ISO 2631-5:2004 had to be revised. In this context, the new version of the ISO 2631-5 published in 2018 has modified the model used to calculate the equivalent of compressive dose, but the health guidance boundaries remained the same as those previously defined in the 2004 version of this standard. However, very limited research has been conducted using the method defined in the ISO 2631-5:2018 standard due to the short time that has elapsed since its publication. Therefore, this study proposes to overcome this research gap. In this sense, the objective of this study is (1) to assess the risk associated with typical operations performed with a HEV, based on the methods defined in ISO 2631-1:1997 and 2631-5:2018; (2) to analyse the methods defined in the standards to determine whether the criteria predict the same level of health risk, and (3) to compare WBV exposure for two drivers with different anthropometric characteristics during operations performed on roads with different surface unevenness.

## 2. Materials and Methods

### 2.1. Measurement of WBV and Field Test

Participants were selected from a convenience sample of professional HEV drivers from companies in the study area. Two drivers were selected with experience in driving HEVs (both with more than 20 years of professional experience in this type of activity), which is especially relevant in the calculation of the adverse effects of cumulative exposure to WBV. In addition, the selected drivers had a significantly different BMI, so that the health effects under two different conditions could be compared (this is a characteristic considered in the method defined in ISO 2631-5:2018) (Table 1). The participants provided information on their work experience (first year of WBV exposure, daily—annual exposure duration and years of WBV exposure).

The HEV selected for the field test was a tractor. Both participating drivers are familiar with the operation of this type of vehicle. The vehicle characteristics are shown in Table 2. According to Regulation No. 167/2013 of the European Parliament and of the Council [40], this vehicle is classified as Class II Category A.

**Figure 1:**
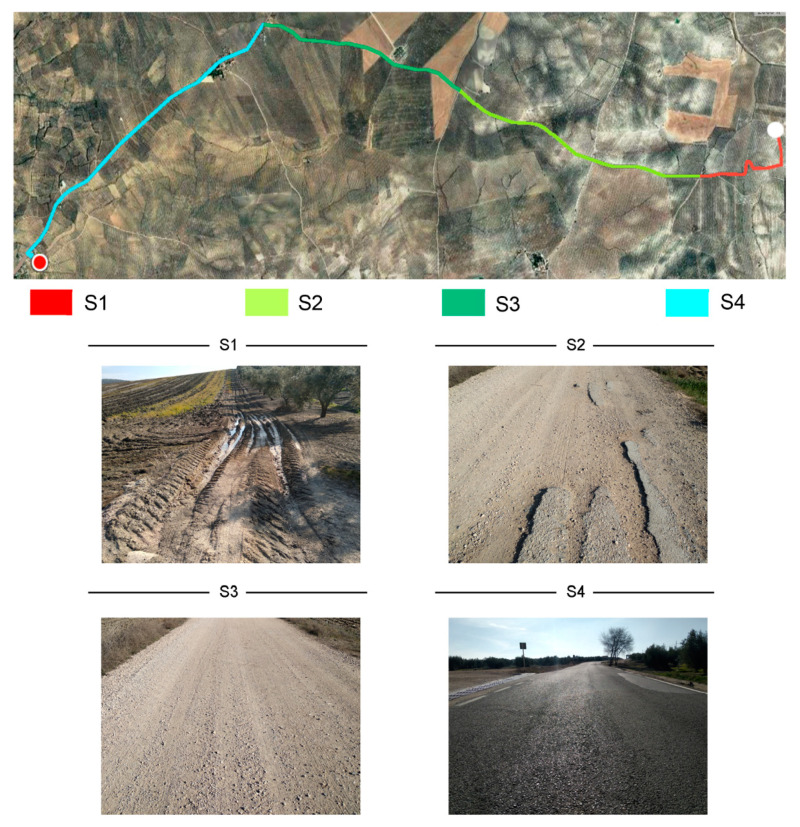
Route selected for field test.

The field test was carried out in Porcuna (Spain) (37°52′11″ N, 4°11′14″ W). Prior to the definition of the test, several drivers of this type of vehicle working in the area were interviewed. Based on the obtained information, a typical work cycle for this type of driver was identified. As a result of this process, a route was selected for the field test. The selected route was representative of the complete work cycle and comprises different types of surfaces. Previous studies have shown that the magnitude of WBV transmitted to the driver is influenced by the characteristics of the road surface [41,42,43]. The route layout and the different surfaces are shown in Figure 1. The route had a length of 10.5 km, comprising 1.5 km off-road, 3 + 2.5 km of unpaved road (two different types were considered) and 3.5 km of tarmac road. The sections of each type of surface were selected to obtain a representative sample of WBV exposure during a working day. This experimental design has been used in previous studies about WBV exposure assessments [9,34,39,44].

**Figure 2:**
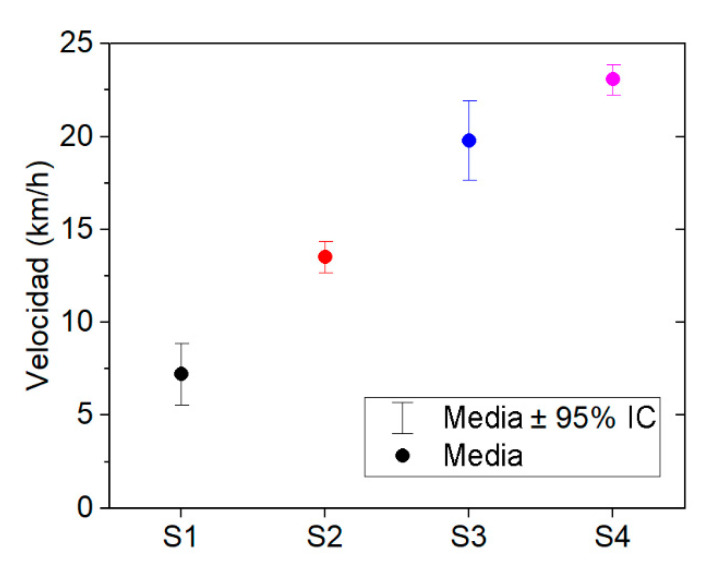
Mean speed of displacement according to type of surface.

Several operational aspects were considered prior to the start of the field test. Tractors are widely used in different operations and these activities often require the use of interchangeable towed machinery or a trailer. In this research, the tractor used a trailer while the measurements were being carried out. Another important aspect considered was the speed. Two factors were considered in order to set the speed limits during the performance of the field test: the maximum forward speed according to the surface conditions and the maximum legal speed allowed for the vehicle on the road. Therefore, in order to characterise the speed on each surface, an experimental campaign was carried out prior to the field test. The obtained results are shown in Figure 2.

On the basis of the above criteria, in the case of off-road driving, the unevenness of this surface requires a limited speed to be maintained in order to ensure the safety of the driver. Consequently, the speed limit was set between 5 and 10 km/h on this type of surface. On unpaved roads, the speed range increases as the surface is more regular than in the previous case. However, since these types of roads can have a wide variety of roughness conditions, this category was divided into two: unpaved road I (high roughness surface), where the speed ranges between 10 and 15 km/h, and unpaved road II (low roughness surface), where the speed ranges between 15 and 25 km/h. Finally, the mean forward speed of the tractor on tarmac roads is higher than on other surfaces (from 20 to 25 km/h). However, it should be noted that the legal speed limit for tractors is 25 km/h when using a trailer or interchangeable towed machinery.

Once the field test procedures were defined, each driver repeated the pre-defined route three times. The test was carried out on weekdays between 8:00 and 10:00 to ensure homogeneous traffic conditions. The set of conditions was the same for the two drivers in order to compare the WBV exposure associated with activities with the same extrinsic factors (including same type of surface, vehicle and forward speed).

### 2.2. Analysis of WBV Exposure

The methods defined in ISO 2631-1:1997 and ISO 2631-5:2018 have been applied in this study in order to assess the level of WBV exposure. Since the aim of this study is to analyse the prediction of health risk associated with HEV activities, methods that assess other types of activities (e.g., high speed marine craft operations) or use other evaluation criteria (e.g., comfort) have not been considered. The procedures of both standards are described in the following sections.

#### 2.2.1. ISO 2631-1:1997

The ISO 2631-1:1997 standard defines the root mean square value A(8) method and the Vibration Dose Value (VDV) method. The A(8) is calculated based on the weighted root mean square acceleration (rms_w_). The rms_w_ value of the weighted acceleration was calculated according to Equation (1). The VDV is calculated based on the fourth power of the weighted acceleration. The vdv_w_ value of the weighted acceleration was calculated according to Equation (2).

(1) $$a_{w}={\left[\frac{1}{T}\overset{T}{\underset{0}{\int }}a_{w}^{2}\left(t\right)\mathrm{dt}\right]}^{1/2}$$

(2) $${\mathrm{vdv}}_{w}={\left[\overset{T}{\underset{0}{\int }}a_{w}^{4}\left(t\right)\mathrm{dt}\right]}^{1/4}$$

where a_w_ is the weighted acceleration at time t (ms^−2^) and T is the duration of the measurement (s). The frequency weighting filter W_d_ is used for the x and y axes, and the W_k_ is used for the z axis.

The value of the daily exposure normalised to 8 h for method A(8) and VDV is calculated according to Equations (3) and (4), respectively.

(3) $$A\left(8\right)=k_{x,y,z}{*\mathrm{rms}}_{w}\sqrt{\frac{T_{\exp}}{T_{0}}}$$

(4) $$\mathrm{VDV}=k_{x,y,z}{*\mathrm{vdv}}_{w}\sqrt[{4}]{\frac{T_{\exp}}{T_{\mathrm{meas}}}}$$

where k denotes the multiplication factor defined for each axis (k_x,y_ =1.4 and k_z_ = 1), T_exp_ is the measurement duration, T_0_ is the reference duration of 8 h, and T_meas_ is the daily duration of vibration exposure.

#### 2.2.2. ISO 2631-5:2018

This standard defines two exposure regimes: one for severe conditions and one for less severe conditions. In this research, the less severe conditions method has been used for the assessment of WBV exposure. This method is indicated for exposures to WBV without free-fall events and where the subject remains seated throughout the measurement. This method uses as input the acceleration measured (x, y and z axes) at the seat surface (minimum). However, accelerations measured at the backrest, feet and hands can also be used.

These accelerations are used to calculate the compressive force between the vertebrae from transfer functions of a biomechanical model. These transfer functions depend on the posture, body mass and body mass index (BMI) of the driver. In addition, the method needs to include the historical vibration exposure (first and last year of exposure, exposure pattern per day and year).

**Figure 3:**
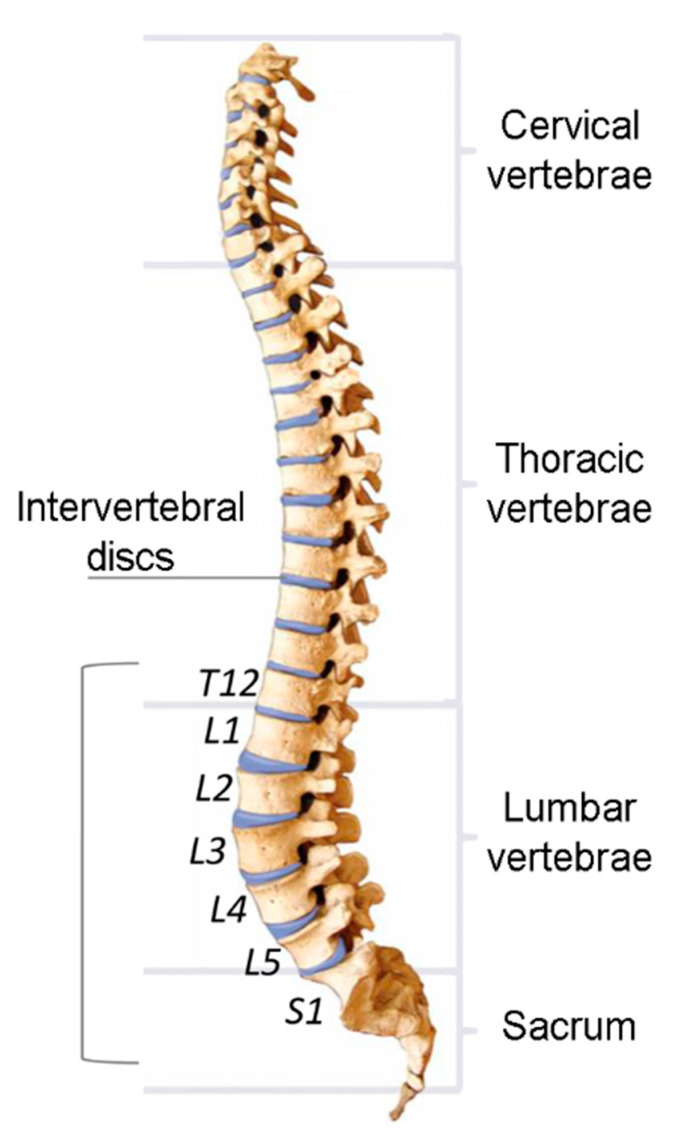
Disc level of the lumbar spine.

The S^A^ (MPa) compression dose is calculated for each disc level of the lumbar spine (T12/L1, L1/L2, L2/L3, L3/L4, L4/L5 and L5/S1, see Figure 3) according to Equation (5):

(5) $$S^{A}={\left[\underset{i}{\sum }{\left(\frac{C_{\mathrm{dyn},i}}{B}\right)}^{6}\right]}^{1/6}$$

where C_dyn,I_ (N) is the sum of the peak compressive forces acting on the vertebral endplate and B (mm^2^) is the area of each disc level of the lumbar spine (T12/L1 = 1460 mm^2^, L1/L2 = 1520 mm^2^, L2/L3 = 1580 mm^2^, L3/L4 = 1590 mm^2^, L4/L5 = 1600 mm^2^ and L5/S1 = 1550 mm^2^).

The equivalent daily compressive dose $S_{d}^{A}$ (MPa) of the lumbar spine is calculated with Equation (6):

(6) $$S_{d}^{A}={\left(\underset{j}{\sum }S_{j}^{A6}\frac{t_{d,j}}{t_{m,j}}\right)}^{1/6}$$

where $S_{j}^{A}$ is the dynamic compressive stress of the lumbar spine due to vibration exposure to condition j, t_d,j_ is the time period of the daily vibration exposure to condition j and t_m,j_ is the time period over which $S_{j}^{A}$ has been measured.

Finally, the risk factor R^A^ for each disc level is calculated according to Equation (7):

(7) $$R^{A}={\left[\overset{n}{\underset{i=1}{\sum }}{\left(\frac{S_{d}^{A}N_{i}^{1/6}}{S_{u,i}^{A}-S_{\mathrm{stat},i}^{A}}\right)}^{6}\right]}^{1/6}$$

where $S_{d}^{A}$ is the constant daily compressive dose; i is the year counted; N_i_ is the number of exposure days per year i; n is the number of exposure years; $S_{u,i}^{A}$ is the ultimate strength of a lumbar vertebra in a person of age (b + i) years, with b being the age at which the exposure started; $S_{\mathrm{stat},i}^{A}$ is the mean value of the compressive‒decompressive force divided by the area of a vertebra endplate B (mm^2^) for year i.

$S_{u,i}^{A}$ is calculated according to Equation (8):

(8) $$S_{u,i}^{A}=6.765024\;\mathrm{MPa}-0.067184\;\left(b+i\right)$$

#### 2.2.3. Health Guidance Caution Zone (HGCZ)

Table 3 shows the limits stated in Directive 2002/44/EC for an eight-hour exposure reference period. ISO 2631-5:2018 defines the limits for the probability of an adverse health effect of exposure to vibration.

### 2.3. Measurement Equipment

The SV38 (SVANTEK) tri-axial seat pad accelerometer was used to measure the acceleration transmitted between the seat and the driver. This accelerometer measures the signal in the x- (front-to-rear), y- (left-to-right) and z- (buttocks-to-head) axes. The acceleration signal was recorded according to the ISO 2631-1:1997 and ISO 2631-5:2018 standard. Additionally, the position of the vehicle was registered by a Global Positioning System (GPS). The signals were post-processed with Matlab^®^ software (MathWorks, Natick, MA, USA).

## 3. Results

### 3.1. Analysis According to ISO 2631-1:1997 and Directive 2002/44/EC

The results obtained using the methods defined in ISO 2631-1:1997 for driver 1 and 2 are shown in Table 4 and Table 5, respectively.

From these results, it can be observed that operations on the S1, S2 and S3 surfaces provide the highest exposure dose for both drivers. It should be noted that, in the case of driver 2, the exposure dose on surfaces S1 and S2 exceeds the EAV limit value stated in Directive 2002/44/EC (A(8) = 0.5 ms^−2^).

Regarding the VDV method, Table 6 and Table 7 show the results obtained on each type of surface for both drivers.

In the case of both drivers, as well as the results obtained from the A(8) method, the level of WBV transmitted is higher on those surfaces with the highest unevenness. The results show that on three surfaces (S1, S2 and S3), the EAV limit established in Directive 2002/44/EC (VDV = 9.1 ms^−1.75^) is exceeded.

The comparison of the WBV assessment provided by both methods, considering each driver and each type of surface, shows that driver 2 has a higher exposure dose than driver 1. According to the results obtained from method A(8), driver 1 was exposed below the HGCZ limits, while driver 2 was exposed above the HGCZ limits for surfaces S1 and S2. In the case of the VDV method, both drivers exceed the EAV limit on all the surfaces except the tarmac road.

### 3.2. Analysis According to ISO 2631-5:2018

This section shows the results obtained using the less severe method defined in ISO 2631-5:2018. The results of the equivalent daily compressive dose $S_{d}^{A}$ for each disc level of the lumbar spine are shown in Table 8 and Table 9 for driver 1 and 2, respectively.

In the case of both drivers, the exposure on all surfaces provides a low probability of adverse health effects. However, as can be seen, the $S_{d}^{A}$ values obtained by driver 1 are higher on all surfaces than those obtained by driver 2.

Regarding the cumulative effect of long-term exposure on the health of drivers, the R^A^ factor has been calculated for each disc level of the lumbar spine. The results obtained are shown in Table 10 and Table 11 for driver 1 and 2, respectively.

**Figure 4:**
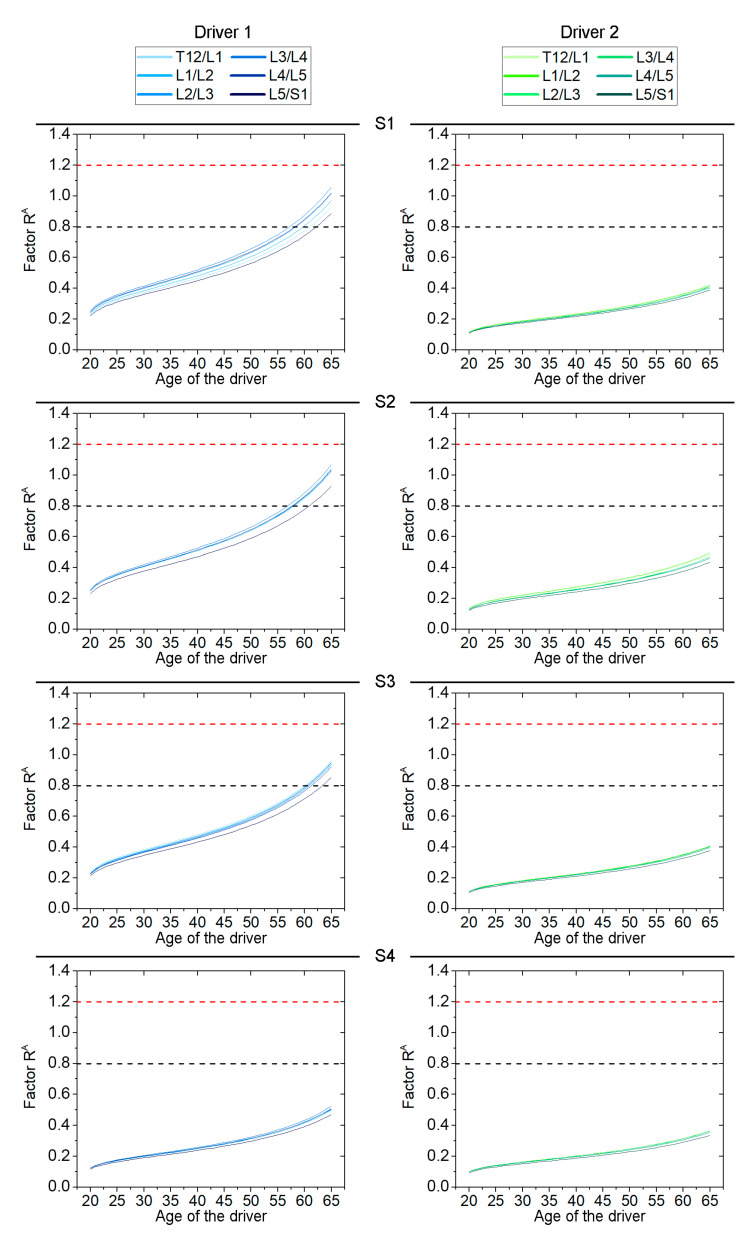
Evolution of the Factor R^A^ at each disc level of the lumbar spine and for each type of surface. Dashed black line indicates low risk probability limit. Dashed red line indicates high probability limit.

The results obtained from the Factor R^A^ indicate that driving on all surfaces provides a low probability of risk associated with WBV exposure, for both drivers (R^A^ < 0.8), for the current year of exposure. However, this method provides a higher R^A^ value for driver 1 than for driver 2 (even taking into account that driver 2 is older and has been exposed for more years than driver 1). In this regard, in order to assume the same years of exposure duration, both drivers were assessed assuming continuous exposure up to the age of 65 years in order to assess long-term exposure under the same conditions. The results obtained are shown in Figure 4.

The results show that driver 1 would exceed the low-risk limit on the three most uneven surfaces. Specifically, he reaches the limit at 58, 57 and 60 years old for the exposures associated with surfaces S1, S2 and S3, respectively. In contrast, the risk probability of driver 2 remains low on all surface types.

## 4. Discussion

**Figure 5:**
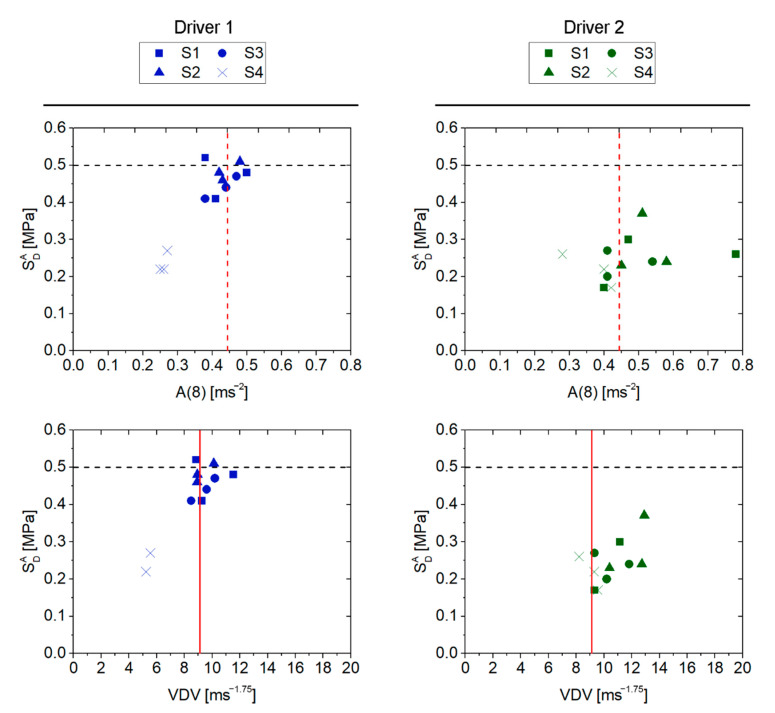
Comparison between results of different WBV assessment methods. Dashed black line indicates low risk probability limit. Dashed red line indicates EAV limit for A(8) method. Solid red line indicates EAV limit for VDV method.

Table 12 shows a summary of the short-term health risk results obtained from the methods defined in ISO 2631-1:1997 and ISO 2631-5:2018, for each type of surface and driver. In addition, a comparison of the obtained results is shown in Figure 5.

Regarding driver 1, an adverse health effect is only indicated by the results obtained from the VDV method on the S1, S2 and S3 surfaces. The other methods did not indicate that the exposure was so detrimental to the health of driver 1, as neither the limit set in Directive 2002/44/EC (in the case of A(8)) nor the limits defined in the ISO 2631-5 standard are reached.

With respect to driver 2, although the VDV method provided the same assessment as for driver 1 (exceeding the VLA limit on the same surfaces), in this case the A(8) method also exceeds the EAV limit for displacements on surfaces S1 and S2. However, the results obtained from $S_{d}^{A}$ indicate that the probability of adverse effects is low in all cases evaluated for driver 2. Indeed, the time that driver 2 would have to be exposed to exceed the $S_{d}^{A}$ low probability limit is more than 24 h in all cases.

Consequently, from these results, it is possible to conclude that the VDV method is more restrictive than the A(8) method. This is mainly due to the fact that the exposures contain shocks to which the VDV method is more sensitive (it is calculated based on the average of the fourth power of the acceleration time history, instead of the second power, which is the method used in the basic A(8) method). Regarding the $S_{d}^{A}$ values obtained, the ISO 2631-5:2018 indicated that the probability is low in all cases evaluated. However, these $S_{d}^{A}$ results (assuming an exposure time of 4 h) are very close to 0.5 MPa in the case of driver 1. In fact, the time that he would have to be exposed to exceed the low probability limit is approximately 6, 5 and 8 h for the driving operations performed on surfaces S1, S2 and S3, respectively. With respect to surface S4, the exposure time would have to be more than 24 h to reach this limit.

Nevertheless, despite the fact that both the VDV and the $S_{d}^{A}$ method aim to assess WBV exposures with multiple shocks, the assessments obtained using both methods provide different valuations. Similar results were found in previous studies by de la Hoz et al. [45] on tractor drivers for the assessment of $S_{d}^{A}$ and Factor R^A^ on different surfaces. Indeed, this fact has been reported by research studies on the comparison of the previous version of the standard (ISO 2631-5:2018) and ISO 2631-1:1997, for the use of HEVs in loading, transport and unloading operations [39], backhoe loaders [6] and railroad locomotives [46,47].

Indeed, previous studies have already suggested that the low and high probability limits for adverse health effects stated in ISO 2631-5:2004 need to be revised [39]. Nevertheless, although the model used to calculate the equivalent compressive dose in ISO 2631-5 has been modified, the limits remain the same as those previously defined in the 2004 version of this standard.

In addition, a study conducted by Bovenzi [16,48] analysed the occurrence of low back symptoms in professional drivers over a two-year period. The study showed a significant association between low back symptoms (low back pain, sciatica) and measures of internal lumbar load ($S_{d}^{A}$ and Factor R^A^ in particular). The results showed that for a 0.1 unit increase in the R^A^ factor, the adjusted risk estimates increased by 28% for low back pain and 32% for sciatica. However, there were no significant associations between low back symptoms and measures of WBV with A(8) and VDV. Moreover, the data showed values associated with lumbar symptom risks below the limit established in ISO 2631-5:2018 associated with a low probability of an adverse health effect (Factor R^A^ < 0. 8). Therefore, the epidemiological study results suggested that the Factor R^A^ limit values indicated by ISO 2631-5:2018 do not sufficiently protect the health of workers exposed to mechanical shocks. However, Bovenzi concluded that further biodynamic and epidemiological studies are needed to validate the results of his study.

This study found that the predicted health risks for short-term exposure assessment according to ISO 2631-1:1997 and ISO 2631-5:2018 provide different criteria. Consequently, since operations may or may not be considered hazardous to the health of HEV drivers depending on the assessment method applied, this may cause confusion to the safety manager during the assessment of the operations. The data presented in this study provide evidence to suggest that the health risk limits should be revised. These results support the findings presented by previous studies about the prior version of the standard. Additionally, it should be noted that less discrepancy was found in the data obtained for driver 1 (high BMI) than for driver 2 (normal BMI) (Figure 5).

Regarding the assessment of long-term WBV exposure, it is worth highlighting the relevance of the cumulative effect of it. Some MSDs, such as low back pain or degenerative spinal disorders, are related to the cumulative effect of WBV exposure. Since Directive 2002/44/CE limits its assessment requirements to short-term assessment, the safety managers would omit the assessment of the long-term effects on workers’ health if they were to follow only the requirements set out in this regulation. Therefore, exposure may be not considered hazardous to the health of the driver in the short term, but may be hazardous in the long-term (as it is observed in the case of driver 1 with a high BMI).

In addition, the results have shown that the anthropometric characteristics of the drivers are a crucial factor in the assessment, as the driver with a high BMI exceeds the lower limit of suffering adverse health effects, in contrast to the driver with a normal BMI who does not reach it (Figure 4). These first results highlight the need to implement WBV health protection programs, especially for drivers with high BMI who belong to a risk group for the development of degenerative spinal disorders. Indeed, the application of the long-term WBV assessment process contributes to achieving one of the objectives’ strategic priorities defined in the European Commission’s Strategic Framework on Health and Safety at Work 2021–2027 (i.e., anticipating and managing change in the context of demographic transitions and improving the prevention of work-related accidents and diseases).

Finally, regarding the limitations of this study, it should be noted that the obtained results do not aim to be generalised or extended to other activities. The data presented in this study have been analysed to compare the health risks obtained with the different assessment methods for drivers with different BMI. Therefore, further research through exposure assessment with ISO 2631-5:2018 is needed to extend the results to other HEVs and operations (i.e., use of interchangeable towed machinery). Additionally, it should be noted that the daily and annual exposures have been assumed to be constant over the working life.

## 5. Conclusions

This study shows a comparison of methodologies for assessing short- and long-term exposure to WBV associated with two HEV drivers with different anthropometric characteristics. Directive 2002/44/EC, ISO 2631-1:1997 and 2631-5:2018 have been used for the assessment. Regarding the short-term WBV exposure assessment, driver 2 (normal BMI) exceeded the EAV for the A(8) method (S1 and S2) and both drivers exceeded the EAV for the VDV method (S1, S2 and S3). These results show the influence of factors such as type of surface and forward speed on the magnitude of WBV exposure. In addition, although the equivalent daily compressive dose $S_{d}^{A}$ (ISO 2631-5:2018) did not exceed the limit of probability of an adverse health effect, recent research has shown that the limits defined in this standard need to be revised. With regard to the long-term WBV exposure assessment, Factor R^A^ results found that only driver 1 would reach the limit (low probability) at 58, 57 and 60 years for exposures associated with surfaces S1, S2 and S3, respectively. This is in contrast to driver 2 (normal BMI) who does not reach this limit. The results showed that the limits established by Directive 2002/44/EC are more restrictive than the limits defined in ISO 2631-5:2018. The discrepancies found in this study were also reported in previous research on the comparison between the limits stated in Directive 2002/44/CE and ISO 2631-5:2004. Although the new version of ISO 2631-5 published in 2018 has modified the assessment model, the limits of suffering adverse health effects remained the same as those previously defined in the ISO 2631-5:2004. The results obtained in this study suggest that these limits still need to be revised, as indicated by the previous studies that analysed the 2004 version.

## Figures and Tables

**Table 1:** Characteristics of drivers.

Driver	D1	D2
Age	48 years	59 years
Height	1.85 m	1.73 m
Weight	120 kg	73 kg
BMI	35.1 kg/m^2^	24.4 kg/m^2^
Mean daily exposure duration to WBV	4 h	4 h
Mean annual exposure duration to WBV	150 days/year	150 days/year
Age at first year of WBV exposure	20 years	20 years
Exposure duration to WBV	28 years	39 years
Posture Group (ISO 2631-5)	3	3

**Table 2:** Characteristics of the vehicle.

Dimensions		Characteristics	
Weight	3800 kg	Power	78 kW
Wheelbase	2.26 m	Cylinder	4
Length	4.19 m	Front tyre	7.50-18
Width	2.05 m	Rear tyre	13.6R38
Height (cab)	2.26 m		

**Table 3:** Health guidance caution zone.

ISO 2631-1:1997/Directive 2002/44/EC			ISO 2631-5:2018		
Exposure Limit Values and Action Value			Probability of an Adverse Health Effect		
Exposure Action Value (EAV)	A(8) = 0.50 ms^−2^	VDV = 9.10 ms^−1.75^	Low	$S_{d}^{A}$ < 0.50 MPa	$R^{A}$ < 0.80
			Moderate	$S_{d}^{A}$ > 0.50 MPa	$R^{A}$ > 0.80
Exposure Limits Value (ELV)	A(8) = 1.15 ms^−2^	VDV = 21.00 ms^−1.75^		$S_{d}^{A}$ < 0.80 MPa	$R^{A}$ < 1.20
			High	$S_{d}^{A}$ > 0.80 MPa	$R^{A}$ > 1.20

**Table 4:** Summary of data obtained from driver 1 according to ISO 2631-1:1997, method A(8).

Surface	No.	rms_wx_ (ms^−2^)	rms_wy_ (ms^−2^)	rms_wz_ (ms^−2^)	A(8)	$\bar{A(8)}$
S1	#1	0.51	0.39	0.49	0.50	0.43
	#2	0.41	0.33	0.47	0.41	
	#3	0.39	0.30	0.45	0.38	
S2	#1	0.37	0.38	0.59	0.42	0.44
	#2	0.37	0.38	0.61	0.43	
	#3	0.36	0.41	0.67	0.48	
S3	#1	0.36	0.39	0.66	0.47	0.43
	#2	0.35	0.40	0.62	0.44	
	#3	0.32	0.34	0.54	0.38	
S4	#1	0.18	0.21	0.38	0.27	0.26
	#2	0.14	0.19	0.36	0.25	
	#3	0.14	0.21	0.36	0.26	

**Table 5:** Summary of data obtained from driver 2 according to ISO 2631-1:1997, method A(8).

Surface	No.	rms_wx_ (ms^−2^)	rms_wy_ (ms^−2^)	rms_wz_ (ms^−2^)	A(8)	$\bar{A(8)}$
S1	#1	0.48	0.43	0.63	0.47	**0.55**
	#2	0.40	0.35	0.38	0.40	
	#3	0.79	0.65	0.56	0.78	
S2	#1	0.36	0.41	0.73	0.51	**0.52**
	#2	0.39	0.46	0.47	0.45	
	#3	0.42	0.59	0.52	0.58	
S3	#1	0.33	0.34	0.58	0.41	0.46
	#2	0.37	0.42	0.40	0.41	
	#3	0.47	0.55	0.52	0.54	
S4	#1	0.22	0.25	0.40	0.28	0.37
	#2	0.27	0.42	0.37	0.42	
	#3	0.20	0.41	0.29	0.40	

* Bold numbers indicate that the value is higher than the EAV.

**Table 6:** Summary of data obtained from driver 1 according to ISO 2631-1:1997, method VDV.

Surface	No.	vdv_wx_ (ms^−2^)	vdv_wx_ (ms^−2^)	vdv_wx_ (ms^−2^)	VDV	$\bar{VDV}$
S1	#1	3.21	2.44	3.56	11.53	**9.88**
	#2	2.27	1.92	3.33	9.27	
	#3	2.36	1.83	2.90	8.82	
S2	#1	1.99	1.96	2.95	8.93	**9.32**
	#2	1.94	1.89	2.89	8.92	
	#3	1.89	1.88	3.12	10.11	
S3	#1	2.17	2.41	3.63	10.20	**9.43**
	#2	2.02	2.32	3.36	9.60	
	#3	1.81	1.99	2.97	8.48	
S4	#1	1.01	1.18	2.14	5.55	5.33
	#2	0.78	1.07	1.99	5.22	
	#3	0.79	1.16	2.00	5.23	

* Bold numbers indicate that the value is higher than the EAV.

**Table 7:** Summary of data obtained from driver 2 according to ISO 2631-1:1997, method VDV.

Surface	No.	vdv_wx_ (ms^−2^)	vdv_wy_ (ms^−2^)	vdv_wz_ (ms^−2^)	VDV	$\bar{VDV}$
S1	#1	3.00	2.68	3.92	11.16	**12.78**
	#2	2.69	2.23	3.63	9.32	
	#3	4.52	3.61	3.05	17.85	
S2	#1	2.61	3.21	5.74	12.90	**12.01**
	#2	1.94	2.46	2.43	10.40	
	#3	3.81	5.06	5.15	12.73	
S3	#1	2.44	2.60	4.30	9.33	**10.45**
	#2	2.42	2.86	2.41	10.20	
	#3	3.51	4.00	4.76	11.81	
S4	#1	2.02	2.10	3.82	8.20	9.01
	#2	2.00	3.00	2.48	9.54	
	#3	1.34	2.40	1.56	9.28	

* Bold numbers indicate that the value is higher than the EAV.

**Table 8:** Summary of data obtained from driver 1 according to ISO 2631-5:2018, less severe method.

Surface	No.	$S_{d}^{A}$						Max. $S_{d}^{A}$	$\bar{S_{d}^{A}}$
		T12/L1	L1/L2	L2/L3	L3/L4	L4/L5	L5/S1		
S1	#1	0.43	0.44	0.46	0.48	0.47	0.43	0.48	0.47
	#2	0.40	0.39	0.40	0.41	0.41	0.37	0.41	
	#3	0.46	0.48	0.50	0.52	0.50	0.45	0.52	
S2	#1	0.47	0.46	0.47	0.48	0.47	0.44	0.48	0.48
	#2	0.44	0.44	0.44	0.46	0.45	0.42	0.46	
	#3	0.51	0.49	0.48	0.49	0.48	0.45	0.51	
S3	#1	0.47	0.45	0.44	0.45	0.44	0.43	0.47	0.44
	#2	0.44	0.43	0.41	0.41	0.41	0.40	0.44	
	#3	0.41	0.41	0.40	0.41	0.40	0.37	0.41	
S4	#1	0.26	0.26	0.26	0.27	0.27	0.26	0.27	0.24
	#2	0.22	0.21	0.21	0.21	0.21	0.20	0.22	
	#3	0.22	0.21	0.21	0.21	0.21	0.20	0.22	

**Table 9:** Summary of data obtained from driver 2 according to ISO 2631-5:2018, less severe method.

Surface	No.	$S_{d}^{A}$						Max. $S_{d}^{A}$	$\bar{S_{d}^{A}}$
		T12/L1	L1/L2	L2/L3	L3/L4	L4/L5	L5/S1		
S1	#1	0.30	0.29	0.28	0.27	0.27	0.26	0.30	0.24
	#2	0.17	0.16	0.15	0.15	0.15	0.15	0.17	
	#3	0.25	0.26	0.25	0.26	0.26	0.25	0.26	
S2	#1	0.37	0.36	0.34	0.33	0.33	0.32	0.37	0.28
	#2	0.23	0.23	0.22	0.22	0.22	0.21	0.23	
	#3	0.23	0.24	0.23	0.24	0.23	0.22	0.24	
S3	#1	0.27	0.26	0.24	0.24	0.23	0.23	0.27	0.24
	#2	0.19	0.19	0.19	0.20	0.19	0.19	0.20	
	#3	0.23	0.24	0.24	0.24	0.24	0.23	0.24	
S4	#1	0.26	0.24	0.23	0.22	0.22	0.22	0.26	0.22
	#2	0.16	0.17	0.16	0.16	0.16	0.15	0.17	
	#3	0.20	0.20	0.22	0.22	0.22	0.20	0.22	

**Table 10:** Summary of data obtained from driver 1 according to ISO 2631-5:2018, Factor R^A^.

Surface	No.	$R^{A}$						Max. $R^{A}$	$\bar{R^{A}}$
		T12/L1	L1/L2	L2/L3	L3/L4	L4/L5	L5/S1		
S1	#1	0.55	0.57	0.60	0.62	0.61	0.54	0.62	0.61
	#2	0.50	0.51	0.52	0.54	0.52	0.47	0.54	
	#3	0.58	0.62	0.65	0.67	0.65	0.57	0.67	
S2	#1	0.59	0.59	0.61	0.62	0.61	0.55	0.62	0.62
	#2	0.56	0.56	0.57	0.59	0.58	0.52	0.59	
	#3	0.65	0.63	0.62	0.63	0.62	0.57	0.65	
S3	#1	0.60	0.58	0.58	0.58	0.57	0.54	0.60	0.56
	#2	0.56	0.55	0.53	0.53	0.53	0.50	0.56	
	#3	0.53	0.53	0.52	0.53	0.51	0.47	0.53	
S4	#1	0.33	0.33	0.34	0.35	0.34	0.32	0.35	0.30
	#2	0.27	0.27	0.27	0.27	0.27	0.25	0.27	
	#3	0.28	0.28	0.27	0.28	0.27	0.26	0.28	

**Table 11:** Summary of data obtained from driver 2 according to ISO 2631-5:2018, Factor R^A^.

Surface	No.	$R^{A}$						Max. $R^{A}$	$\bar{R^{A}}$
		T12/L1	L1/L2	L2/L3	L3/L4	L4/L5	L5/S1		
S1	#1	0.34	0.33	0.32	0.31	0.31	0.30	0.34	0.28
	#2	0.19	0.18	0.18	0.18	0.17	0.17	0.19	
	#3	0.28	0.30	0.29	0.30	0.29	0.28	0.30	
S2	#1	0.42	0.41	0.38	0.38	0.37	0.36	0.42	0.32
	#2	0.26	0.27	0.25	0.25	0.25	0.23	0.27	
	#3	0.26	0.27	0.26	0.27	0.27	0.24	0.27	
S3	#1	0.30	0.29	0.28	0.27	0.27	0.26	0.30	0.27
	#2	0.21	0.22	0.22	0.22	0.22	0.21	0.22	
	#3	0.26	0.27	0.27	0.28	0.27	0.26	0.28	
S4	#1	0.29	0.27	0.26	0.25	0.25	0.25	0.29	0.25
	#2	0.19	0.19	0.19	0.19	0.18	0.17	0.19	
	#3	0.22	0.23	0.25	0.26	0.25	0.23	0.26	

**Table 12:** Summary of health risk results obtained by ISO 2631-1 and ISO 2631-5.

Surface	Driver 1	VDV (ms^−1.75^)	$S_{d}^{A}(\mathrm{MPa})$	Driver 2	VDV (ms^−1.75^)	$S_{d}^{A}(\mathrm{Mpa})$
	A(8) (ms^−2^)			A(8) (ms^−2^)		
S1	0.43	**9.88**	0.47	**0.55**	**12.78**	0.24
S2	0.44	**9.32**	0.48	**0.52**	**12.01**	0.28
S3	0.43	**9.43**	0.44	0.46	**10.45**	0.24
S4	0.26	5.33	0.24	0.37	9.01	0.22

* Bold numbers indicate that the value is higher than the EAV.

## Data Availability

Data are provided upon request to the corresponding author.
